# Vermont Hospital for the Insane at Brattleboro’

**Published:** 1863-03

**Authors:** 


*Vermont Hospital for the Insane at Brattleboro'.*—A large portion of the building of this institution was, we regret to learn, destroyed by fire on the 21st of December last. The patients were all saved.

